# Small-Molecule Polθ Inhibitors Provide Safe and Effective Tumor Radiosensitization in Preclinical Models

**DOI:** 10.1158/1078-0432.CCR-22-2977

**Published:** 2023-01-23

**Authors:** Gonzalo Rodriguez-Berriguete, Marco Ranzani, Remko Prevo, Rathi Puliyadi, Nicole Machado, Hannah R. Bolland, Val Millar, Daniel Ebner, Marie Boursier, Aurora Cerutti, Alessandro Cicconi, Alessandro Galbiati, Diego Grande, Vera Grinkevich, Jayesh B. Majithiya, Desiree Piscitello, Eeson Rajendra, Martin L. Stockley, Simon J. Boulton, Ester M. Hammond, Robert A. Heald, Graeme C.M. Smith, Helen M.R. Robinson, Geoff S. Higgins

**Affiliations:** 1 Department of Oncology, University of Oxford, Oxford, United Kingdom.; 2 Artios Pharma, Babraham Research Campus, Cambridge, United Kingdom.; 3 Target Discovery Institute, Nuffield Department of Medicine, University of Oxford, Oxford, United Kingdom.; 4 The Francis Crick Institute, London, United Kingdom.

## Abstract

**Purpose:**

DNA polymerase theta (Polθ, encoded by the *POLQ* gene) is a DNA repair enzyme critical for microhomology mediated end joining (MMEJ). Polθ has limited expression in normal tissues but is frequently overexpressed in cancer cells and, therefore, represents an ideal target for tumor-specific radiosensitization. In this study we evaluate whether targeting Polθ with novel small-molecule inhibitors is a feasible strategy to improve the efficacy of radiotherapy.

**Experimental Design:**

We characterized the response to Polθ inhibition in combination with ionizing radiation in different cancer cell models *in vitro* and *in vivo*.

**Results:**

Here, we show that ART558 and ART899, two novel and specific allosteric inhibitors of the Polθ DNA polymerase domain, potently radiosensitize tumor cells, particularly when combined with fractionated radiation. Importantly, noncancerous cells were not radiosensitized by Polθ inhibition. Mechanistically, we show that the radiosensitization caused by Polθ inhibition is most effective in replicating cells and is due to impaired DNA damage repair. We also show that radiosensitization is still effective under hypoxia, suggesting that these inhibitors may help overcome hypoxia-induced radioresistance. In addition, we describe for the first time ART899 and characterize it as a potent and specific Polθ inhibitor with improved metabolic stability. *In vivo*, the combination of Polθ inhibition using ART899 with fractionated radiation is well tolerated and results in a significant reduction in tumor growth compared with radiation alone.

**Conclusions:**

These results pave the way for future clinical trials of Polθ inhibitors in combination with radiotherapy.

**Translational Relevance**

Radiotherapy plays a key role in the curative management of many solid tumors. However, treatment failures can occur due to the intrinsic capability of cancer cells to survive ionizing radiation, tumor microenvironmental factors such as hypoxia, and limitations in the maximum radiation dose that can be delivered before normal tissues become damaged. Polθ, a DNA polymerase involved in DNA damage repair, represents an ideal target for improving radiotherapy outcomes, as it is frequently expressed in cancer cells but is mostly absent in normal tissues. We demonstrate that pharmacologic Polθ inhibition impairs ionizing radiation-induced DNA damage repair and radiosensitizes human cancer cells under hypoxic conditions that are found in patient tumors. Importantly, we demonstrate that Polθ inhibition provides effective tumor radiosensitization in mouse xenografts at a clinically relevant radiotherapy fractionation schedule. Our study will encourage the clinical development of this class of small-molecule Polθ inhibitors in combination with radiotherapy.

## Introduction

Approximately 50% of patients with cancer receive radiotherapy yet, despite technical improvements in delivery, survival rates following radical radiotherapy remain poor for many tumor types (1, 2). In addition, the radiation delivered to adjacent tissues is still frequently associated with significant side-effects. One strategy to improve radiotherapy outcome is to increase the radiosensitivity of tumor cells without affecting the sensitivity of the surrounding normal cells (3). DNA polymerase theta (Polθ), a DNA repair enzyme that has low or absent expression in most normal tissues, but which is frequently overexpressed in many cancer types, represents an ideal tumor-specific radiosensitization target (4–8).

Polθ, encoded by the *POLQ* gene, plays a key role in microhomology-mediated end-joining (MMEJ; refs. 9–12), a DNA double-strand break (DSB) repair pathway that depends on the presence of short homologous sequences (2–4 bp) across the break site. MMEJ has often been described as a back-up pathway for nonhomologous end-joining (NHEJ) or repair by homologous recombination (HR; ref. 13), but in specific circumstances MMEJ can function when both NHEJ and HR are active (14–16). Similarly to HR, MMEJ requires 5′-3′ resection of the DNA break, but during MMEJ the resection is much shorter and serves to reveal small microhomologies between the two single-stranded DNA (ssDNA) strands to enable their annealing (17, 18). In contrast to HR—which is broadly considered error-free—MMEJ is an error-prone DSB repair pathway associated with deletions and introduction of single-base errors (19).

Polθ deficiency is synthetically lethal with defects in HR repair, suggesting that cancer cells with deficiency in HR, for example through BRCA mutations, become more reliant on the MMEJ pathway to repair DSBs and potentially other lesions (7, 20). However, we have also shown that depleting Polθ through siRNA radiosensitizes tumor cells without mutations in BRCA genes, which suggests that Polθ inhibition may provide radiosensitization for a wide range of tumors, irrespective of HR status (21). Therefore, we initiated a Polθ inhibitor discovery program and recently described the identification of novel and specific Polθ inhibitors, including ART558 (22). These compounds, which specifically inhibit the polymerase activity of Polθ, were shown to be synthetically lethal with BRCA and Shieldin deficiency (22).

Here, we show that small molecule inhibitors targeting Polθ effectively radiosensitize tumor cells *in vitro* and *in vivo* and reveal their effect under hypoxia, a feature frequently associated with solid tumors and linked to radioresistance. Our results evidence that the radiosensitization induced by Polθ inhibition is cell-cycle–dependent and caused by defects in DSB repair and increased genomic instability. We also report for the first time ART899 as a specific and potent Polθ inhibitor with improved *in vivo* stability. Combined with fractionated radiation, Polθ inhibition exhibits effective tumor growth delay in mouse xenografts and, importantly, this combination treatment is well tolerated.

## Materials and Methods

### Cell culture and reagents

H460 (RRID:CVCL_0459), HCT116 (RRID:CVCL_0291), T24 (RRID:CVCL_0554), HeLa (RRID:CVCL_0030), MRC-5 (RRID:CVCL_0440), HEK-293 (RRID:CVCL_0045), and HIEC-6 (RRID:CVCL_6C21) cells were purchased from ATCC; AG01552 cells were obtained from the Coriell Institute. MDA-MB-436 (RRID:CVCL_0623), DLD-1 (RRID:CVCL_0248), and DLD-1 BRCA2 KO were obtained and grown as described previously (22). Cells were authenticated by short tandem repeat (STR) profiling (carried out by LGC standards) if grown for more than 6 months accumulatively after acquisition. U2OS Polθ KO cells were generated CRISPR/Cas9 technology by Synthego for Artios, which also provided the U2OS WT cells (RRID:CVCL_0042). Cells were grown in RPMI (H460, T24), DMEM (HCT116, HeLa), MEM (MRC-5, AG01552), or McCoy's medium (U2OS), all supplemented with 10% FBS and incubated at 37°C and 5% CO_2_. All these media were purchased from Sigma-Aldrich/Merck. HIEC-6 cells were grown in OptiMEM (Gibco) supplemented with 20 mmol/L HEPES (Sigma-Aldrich/Merck), 10 mmol/L Glutamax (Gibco), 10 ng/mL EGF (PeproTech), and 4% FBS. FBS was purchased from Life Technologies and the same serum batch was used for all experiments. Regular testing with MycoAlert Kit (Lonza) confirmed the absence of mycoplasma contamination. ART558 was produced as described previously (22). ART899 was produced as described for its nondeuterated form, ART812 (22), with the addition of deuterium during synthesis. Compounds were stored as powder under vacuum at room temperature in the dark. Compounds were dissolved in DMSO at 12 mmol/L and these stocks dissolved were kept at room temperature in the dark.

### Clonogenic survival experiments

Cells were plated as single cells in 6-well plates or 24-well plates and left to settle for a minimum of 5 hours before treatment. Seeding densities were optimized for each cell line; increasing cell numbers were used for higher ionizing radiation (IR) doses to account for cell death. One hour prior to IR, a concentrated working stock of ART558 or ART899 was prepared in medium and added to the cells to yield a final concentration of 1 or 3 μmol/L. Cells were irradiated in a cesium-137 irradiator (GSR D1 from Gamma Service; dose rate 1.2 Gy/min). For the fractionated radiation experiments, 2 Gy fractions were delivered every 24 hours. Compound was removed by performing a medium change 3 days after IR (for the fractionated radiation experiments, 3 days after the last IR dose). Colonies were grown for 8 to 14 days, stained with crystal violet, and counted using the Gelcount automated colony counter (Oxford Optronics). The plating efficiency (PE = average colony number/cells plated) and the surviving fraction (SF = PE_IR Dose_/PE_0 Gy_) at a given IR dose was calculated. Survival data were fitted according to a linear quadratic equation and, for comparison between curves, the sensitization enhancement ratio at a surviving fraction of 0.10 (SER_10_) was calculated. The oxygen enhancement ratio (OER) was defined as the ratio between the radiation doses at 10% survival of hypoxic and normoxic cells.

### Hypoxia experiments

Hypoxia experiments were performed in a BactronEZ anaerobic chamber (Shel Lab) for concentrations of <0.1% O_2_ or a M35 hypoxia workstation (Don Whitley Ltd.) for concentrations of 0.5% O_2_. Both chambers were humidified and kept at 37°C. Cells were left to settle at 37°C in a standard (normoxic) 37°C incubator for a minimum of 6 hours before transfer into the hypoxia chamber and were then left inside the chamber for at least 14 hours prior to IR. To perform radiations under hypoxic conditions, culture plates were transferred into airtight Perspex boxes that had also been placed inside the hypoxia chamber overnight (23). The boxes containing the culture plates were then placed inside the cesium irradiator for irradiation, thus maintaining hypoxia during irradiation. After radiation, culture plates were removed from the Perspex boxes and returned to a normoxic 37°C incubator for colonies to grow.

### Alamar blue assay

Alamar blue is a resazurin-based, nontoxic, cell-permeable compound, which becomes fluorescent when it is metabolized by viable cells, allowing to assess the relative quantity of viable cells. Cells were seeded in 96-well plates, left to attach overnight, and then treated with 3 μmol/L ART899 and 5×2 Gy of IR (six replicate wells per condition) as described above for clonogenic survival experiments. Eight days after the first IR fraction, cells were incubated with 15 μg/mL alamar blue (Sigma-Aldrich/Merck), diluted in the medium, for 4 hours in an incubator at 37°C. Fluorescence was measured using a PolarStar Omega plate reader (BMG Labtech).

### Transfection

A reverse transfection procedure was carried using RNAiMax (Invitrogen) following manufacturer instructions. The Silencer Select (Life Technologies) nontargeting siRNA (siNT) and the siRNA targeting *POLQ* (sense strand sequence: CCGCUUUUGGAGUCAGUAA) were used at a concentration of 40 nmol/L. Treatments were initiated 72 hours after transfection. Knockdown was confirmed by Western blotting in cells collected at the time of IR.

### Western blotting

Cells were washed in PBS and lyzed in RIPA buffer (Thermo Fisher Scientific) supplemented with protease inhibitors (Roche) and benzonase (Merck). Lysates were cleared by centrifugation and protein content was quantitated using the Bicinchoninic acid assay (BCA; Thermo Fisher Scientific). Equal amounts of samples (50–100 μg) were resolved by SDS-PAGE in 3% to 8% Tris-Acetate gels (Thermo Fisher Scientific). Gels were wet-transferred in 1x NuPAGE Transfer Buffer (Thermo Fisher Scientific) 20% ethanol and 0.05% SDS to nitrocellulose membranes (Millipore). Membranes were then blocked in 5% Milk/Tris-buffered saline + 0.01% Tween-20 (TBST), which was also used for subsequent incubation steps. Membranes were probed overnight at 4°C with antibodies against Polθ (rabbit polyclonal, raised against a GST-fusion protein encoding residues 1290–1389, Artios) and vinculin (mouse monoclonal; Santa Cruz Biotechnology, Catalog No. sc-73614, RRID:AB_1131294). The membranes were then washed with TBST and incubated with HRP-conjugated anti-rabbit IgG secondary antibody (Thermo Fisher Scientific) and with IRDye 800CW Goat anti-mouse IgG Secondary antibody (Li-COR) for 1 hour at RT in the dark. After washing twice in TBST, luminescent signals (from rabbit antibodies) were detected by ECL detection reagent (Thermo Fisher Scientific) and imaged on an Amersham Imager 600RGB; the fluorescent signals (from mouse antibodies) were detected using the Odyssey M Imager (Li-COR).

### Cell-cycle analysis of cells subjected to fractionated IR

HCT116 cells were seeded in 6-well plates and treated according to the different experimental arms: vehicle (DMSO), 10 μmol/L ART558, 3 × 2 Gy of IR (each fraction delivered every 24 hours), and 10 μmol/L ART558 combined with 3 × 2 Gy of IR. ART558 treatment was started 1 hour before the first fraction of IR and maintained throughout the whole duration of the experiment. Approaching each timepoint of analysis, cells were labelled with 10 μmol/L 5-ethynyl-2′-deoxyuridine (EdU) for 1 hour, and then fixed in ice-cold 70% ethanol in PBS and kept at −20 °C overnight. EdU was detected using the Click-iT Plus Kits (Life Technologies). Cells were then incubated with FxCycle violet. Data were acquired using a flow cytometer (BD LSRFortessa) and analyzed using FlowJo (BD Biosciences, RRID:SCR_008520). The gating strategy to identify the different phases of cell cycle is indicated in Supplementary Fig. S2A.

### Double thymidine (DT) block experiment

HeLa cells were plated as single cells for clonogenic survival assays, left to settle for 5 hours and then incubated with 2 mmol/L thymidine for 15 hours, released for 9 hours after a PBS wash and medium replacement, and incubated again with thymidine for 15 hours. Then, cells were either released immediately after exposure to IR, or irradiated at 6 hours after release. In all cases, either vehicle (DMSO) or ART558 was added 1 hour before IR. To confirm the cell-cycle position of the synchronized cells at the time of IR exposure, the same DT block protocol was applied in parallel to cells that were subsequently fixed in 70% ice-cold ethanol for DNA content analysis. Fixed cells were incubated with 50 μg/mL propidium iodide and 200 μg/mL RNase in PBS for 20 minutes at 37°C, and then analyzed using a Cytoflex (Beckman Coulter) cytometer. After exclusion of doublets, the distribution of cells according to their DNA content (PI intensity) was determined using FlowJo (BD Biosciences) software.

### Nanoluciferase-based MMEJ assay

The nanoluciferase-based MMEJ assay was carried out following a similar protocol to what has been described previously (22). Briefly, the Nano-luciferase MMEJ reporter construct (a linearized plasmid with protruding microhomology ssDNA ends engineered such to recombine into a functional luciferase construct only after correctly performed MMEJ; ref. 22) was transfected into cells together with a control firefly luciferase plasmid. Transfected cells were plated into 384- or 96-well plates already containing diluted compound and luminescence was read 24 hours after transfection. Firefly and nano-luciferase levels were detected using the Nano-Glo Dual-Luciferase Reporter Assay Kit (Promega) and luminescence was measured with a Clariostar plate reader (BMG Labtech). The nanoluciferase signal was normalized to the firefly luciferase signal to correct for cell density and transfection efficiency, and finally normalized to vehicle-treated controls.

### Immunofluorescence staining for DNA damage foci and micronuclei

Cells were seeded either into 96-well thin bottom imaging plates (Perkin Elmer; for micronuclei analysis) or onto round coverslips in 24-well plates (for foci analysis) and left to settle for a minimum of 6 hours at 37°C before treatment. ART558 was added 1 hour prior to IR and cells were irradiated as described above. At specific time points after IR, cells were fixed in 4% paraformaldehyde for 15 minutes at room temperature. Cells were permeabilized in PBS with 1% BSA, 0.5% Triton, and 1% goat serum for at least 30 minutes. Cells were then incubated overnight at 4°C using any of the following primary antibodies diluted in PBS/1% BSA: rabbit anti-RAD51 (Cell Signaling Technology, Catalog No. 8875, RRID:AB_2721109; 1/100), rabbit anti-γH2AX (Novus, Catalog No. NB100–2280, RRID:AB_10000580; 1/2,000), mouse anti-53BP1 (Cell Signaling Technology, Catalog No. 4937, RRID:AB_10694558; 1/1,000), or mouse anti-dsDNA (to visualize micronuclei; Santa Cruz Biotechnology, Catalog No. sc-58749, RRID:AB_783088; 1:800). Following three PBS washes, cells were incubated for 90 minutes at room temperature with secondary antibodies diluted in PBS/1% BSA: Alexa Fluor 488 goat anti-rabbit (Life Technologies, #A11070; 1/1200) or Alexa Fluor 594 goat anti-mouse (Life Technologies, #A11020; 1/1,200). DAPI (Sigma-Aldrich/Merck, #D9542) was also added at 0.5 μg/mL final concentration at this point. Following three PBS washes, plates were imaged using an automated confocal microscope (GE Healthcare IN Cell 6000), whereas coverslips were mounted onto slides using ProLong Diamond antifade mountant (Thermo Fisher Scientific) and imaged using a Nikon NiE microscope. Foci were assessed using the image analysis software Imaris (Oxford Instruments).

### Microsome assay

Metabolic stability of compounds in mouse and rat liver microsomes was conducted at WuXi AppTec Co. The compounds (ART558 and ART899) and controls (testosterone, diclofenac, and propafenone) diluted in 100 mmol/L potassium phosphate buffer (pH 7.4) were mixed with either mouse or rat microsome solution (Xenotech) at a 1:10 volume ratio (final concentration of compounds and microsomes, 1 μmol/L and a 0.5 mg protein/mL, respectively), and incubated for 10 minutes at 37°C. NADPH was then added to start the reactions. At different time intervals, three fourths of the final volume cold acetonitrile (containing 100 ng/mL tolbutamide and 100 ng/mL labetalol as internal standards) was added to stop the reaction. After centrifugation, the supernatants were analyzed by LC/MS-MS. A first-order kinetics equation was used to calculate the half-life (*t*_1/2_), which was in turn used to calculate the intrinsic clearance based on the presence of the parent compound, according to the following equation: CLint = (0.693 / *t*_1/2_) × (1 / mg × mL^−1^ microsomal protein in reaction system) (μL × min^−1^ × mg^−1^), as described previously (24).

### *In vivo* studies

For the pharmacokinetics study, Nu/Nu mice (6–8 weeks) were dosed orally for 11 days with ART899 dissolved in 5% DMSO, 5% ethanol, 20% TPGS, 30% PEG400, 40% water. Plasma samples were collected at various time points and ART899 levels were determined by CEMAS using standard protocols with a calibration curve and LC/MS-MS (API 5500) readout, and the free (nonprotein bound) fraction of inhibitor in plasma was calculated. The pharmacokinetics study was carried out by WuXi AppTec.

The *in vivo* efficacy study was performed by Charles River. For the efficacy study, HCT116 cells (5 × 10^6^ in 100 μL PBS) were injected subcutaneously into female athymic nude mice [Crl:NU(NCr)-Foxn1nu, 8–12 weeks old; RRID:IMSR_CRL:490]. Fifteen days after tumor implantation, when average tumor size approached the target range of 80 to 180 mm^3^—designated as day 1 of the study—tumor-size-matched animals (average tumor size 120.6–121.4 mm^3^) were sorted into the four treatment groups: vehicle, ART899, 10 × 2 Gy + vehicle, and 10 × 2 Gy + ART899. Mice were treated with 150 mg/kg ART899 (dissolved as above) given by oral gavage twice a day for 12 days. Radiation was given on days 1–5 and 8–12 at 2 Gy per fraction targeted to the tumor while the mice were under isoflurane anesthesia. Tumor size and weight measurements were taken by one investigator not blinded to treatment group. Animals were weighed daily on days 1 to 5, then twice per week until the completion of the study. The endpoint for each group was a mean tumor size of 1,500 mm^3^ or a maximum of 49 days. As such, the unirradiated treatment arms (vehicle or ART899 alone) were terminated at day 21, the 10 × 2 Gy arm on day 39, and the combination arm on day 49. The mice were observed frequently for overt signs of any adverse, treatment-related side effects, and clinical signs were recorded when observed. Any animal with weight loss exceeding 30% for one measurement, or exceeding 25% for three measurements, was euthanized as a treatment-related death. Acceptable toxicity was defined as a group mean body weight loss of less than 20% during the study and no more than 10% treatment-related death. Clinical observations (including necropsies of main organs, especially spleen, liver, and lymph nodes) were recorded at the time all available animals in each group were sampled. In the efficacy study, we did not observe any treatment-related death, nor significant decrease of body weight. In particular, a minimal reduction in body weight was associated to the IR treatment, and the group treated with IR + ART899 displayed a mean and distribution of body weight comparable with the group treated with IR alone. Necropsy analysis did not evidence any abnormality associated to the IR + ART899 combined treatment.

For the pharmacokinetics study, procedures related to animal handling, care, and experimental treatments were performed according to the guidelines approved by the Institutional Animal Care and Use Committee (IACUC) of WuXi AppTec Co. following the guidance of the Association for Assessment and Accreditation of Laboratory Animal Care (AAALAC). The efficacy study performed by Charles River complied with the recommendations of the Guide for Care and Use of Laboratory Animals with respect to restraint, husbandry, surgical procedures, feed and fluid regulation, and veterinary care. The animal care and use program at Charles River Discovery Services is accredited by AAALAC.

### Statistical analysis

Results are shown as the mean ± SD of a minimum of three observations unless otherwise indicated in the figure legends. Two-tailed *t* tests were used to calculate statistical significance unless indicated otherwise; a *P* value of <0.05 was considered statistically significant. The linear quadratic model, *S* = exp(*αD* – *βD*^2^), was used to fit clonogenic survival graphs, with *S* denoting survival and *D* the IR dose in Gy. Graphpad Prism (RRID:SCR_000306) was used for all statistical calculations and curve fitting.

### Data availability statement

Any of the datasets that have been analyzed for the manuscript will be shared upon request.

## Results

### ART558 induces Polθ-dependent radiosensitization in cancer cell lines

**Figure 1.**
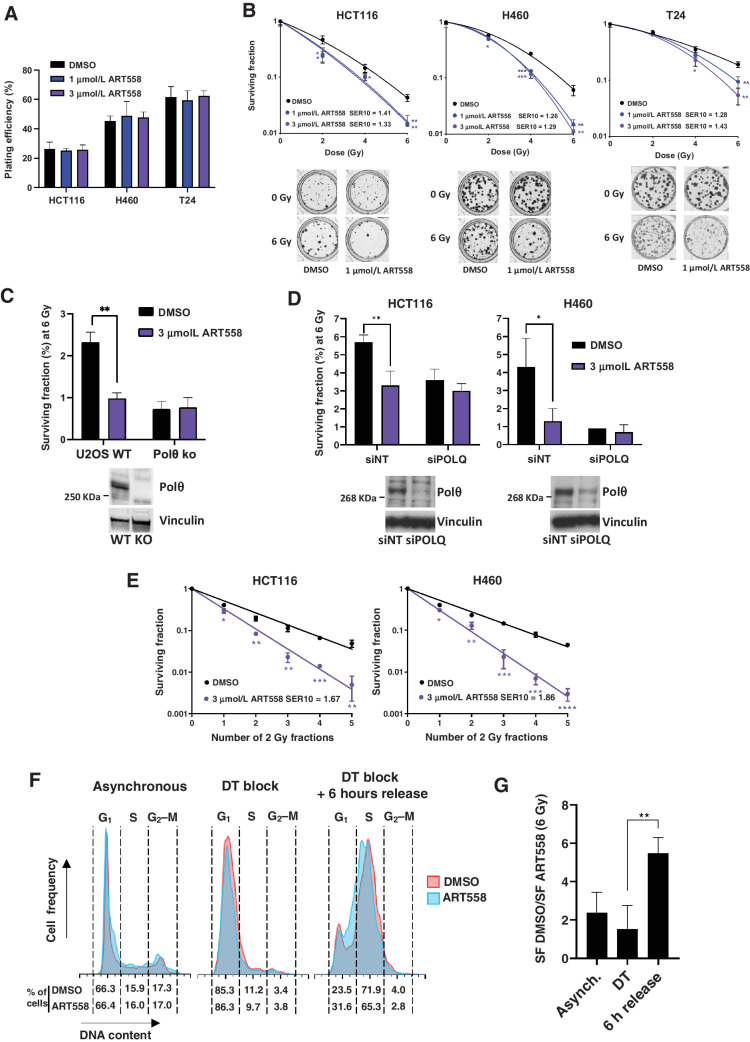
The Polθ inhibitor ART558 radiosensitizes tumor cells. **A** and **B,** Clonogenic survival of HCT116, H460, and T24 cells treated with ART558 and/or IR. **A,** Plating efficiency for unirradiated cells. **B,** Surviving fractions as a function of the irradiation dose. Representative wells for 0 Gy and 6 Gy ± 1 μmol/L ART558 are shown for each cell line. **C,** Clonogenic survival of U2OS WT and Polθ KO cells treated with 3 μmol/L ART558 and 6 Gy IR. Bar graphs show the surviving fraction at 6 Gy. The Western blot insets confirm the lack of Polθ expression in the U2OS Polθ KO cells. **D,** Clonogenic survival of HCT116 and H460 cells transfected with either a control, nontargeting siRNA (siNT) or an siRNA targeted against *POLQ* (si*POLQ*) and treated with 3 μmol/L ART558 and 6 Gy IR. Bar graphs show the surviving fraction at 6 Gy. Western blots show Polθ expression at the time of irradiation. **E,** Clonogenic survival of H460 and HCT116 treated with 3 μmol/L ART558 and 5×2 Gy (2 Gy once per day for 1 to 5 days). **F** and **G,** Clonogenic survival following irradiation and treatment with 3 μmol/L ART558 of synchronized HeLa cells. **F,** Representative histograms showing the cell-cycle distribution at the time of IR (synchronized in G_1_ by DT block or after 6 hours release from DT block, compared with asynchronous cultures). **G,** Degree of radiosensitization estimated by the ratio between the surviving fraction of DMSO- and ART558-treated cells after IR (SF DMSO / SF ART558). Data correspond to average ± SD from three independent experiments (*, *P* < 0.05; **, *P* < 0.01; ***, *P* < 0.001; ****, *P* < 0.0001).

We have recently developed ART558, a new and highly specific Polθ inhibitor which displayed synthetic lethality with BRCA loss (22, 25, 26). In this study, we investigated whether Polθ inhibition could serve as an anticancer therapeutic agent beyond the context of BRCA deficiency, in combination with radiotherapy. We first assessed the effect of ART558, alone and in combination with IR, in three tumor cell lines that do not harbor mutations in BRCA-1 or BRCA-2 genes (27): HCT116 (colorectal), H460 (lung), and T24 (bladder). We selected these cell models because they form quantifiable colonies, are commonly used in radiotherapy studies (28, 29), are derived from tumor types relevant for radiotherapy, and express average *POLQ* levels (Supplementary Fig. S1; ref. 27). Using colony formation assays we confirmed that ART558 does not cause any effect in the absence of IR (Fig. 1A), but effectively radiosensitizes all three BRCA-proficient cell lines from 1 μmol/L concentration [Fig. 1B; sensitization enhancement ratio at 10% survival (SER_10_) > 1.2 at 1 μmol/L ART558 in all three cell lines].

Because 3 μmol/L gave a comparable or stronger response than 1 μmol/L while not affecting viability without radiation, this ART558 concentration was chosen for subsequent experiments. U2OS Polθ knockout (KO) cells were more sensitive to IR than parental wild-type (WT) U2OS cells and were not radiosensitized by ART558, whereas the WT U2OS cells were significantly radiosensitized, confirming that the radiosensitizing effect of ART558 is Polθ-specific (Fig. 1C). Similarly, ART558 did not further radiosensitize HCT116 and H460 cells upon siRNA-mediated Polθ depletion, confirming the radiosensitizing activity of ART558 is also Polθ-specific in these cell lines (Fig. 1D). Since radiotherapy is given over multiple fractions in clinical settings (30), we tested ART558 combined with fractionated radiotherapy, and showed that the radiosensitizing effect of ART558 increases with the number of fractions (Fig. 1E). For H460 cells there was up to a 14-fold decrease in survival with 5 × 2 Gy in the presence of ART558 compared with IR alone. For HCT116 cells, this factor was up to 10-fold.

### Cell-cycle–related effects of Polθ inhibition by ART558 in combination with radiation

As MMEJ functions mainly during the S and G_2_ cell-cycle phases (31), we hypothesized that Polθ inhibition would have a greater effect in these cell-cycle phases. To investigate this, we used the cervical cancer cell line HeLa due to its suitability for cell-cycle synchronization using DT block. As expected, we found that HeLa cells irradiated when they are mostly traversing S phase are radiosensitized by ART558 to a higher extent than cells irradiated whilst synchronized in G_1_ (Fig. 1F and G). Because fractionated radiotherapy allows for cells to move to different cell-cycle phases where they might be more sensitive to Polθ inhibition (30), this finding may partly explain our observation that ART558-mediated radiopotentiation is higher with multiple IR fractions.

To address whether ART558 could impact the cell-cycle distribution at the time of the radiation fractions—which may also contribute to the enhanced radiosensitization with the fractionated schedule—we analyzed the proportion of cells in the different cell-cycle phases in HCT116 cells subjected to fractionated IR, at the time of the third and fourth radiation fractions (Supplementary Fig. S2). Compared with IR alone, ART558 caused a decrease in actively replicating S phase cells and an increase in polyploid cells (Supplementary Fig. S2B), the latter indicative of cell senescence. The small variation in the proportion of actively replicating S phase cells in relation to the whole cell population (a decrease of 12% and 7% at the third and fourth fractions, respectively; Supplementary Fig. S2B), together with the fact that cells in S phase are inherently less radiosensitive than cells in G_1_ but potentially more susceptible to ART558-mediated radiosensitization, suggest that differences in the cycle distribution at the time of radiation do not play a major role in the radiosensitizing effect of ART558 when combined with a fractionated schedule.

### ART558 treatment impairs IR-induced DNA damage repair

**Figure 2.**
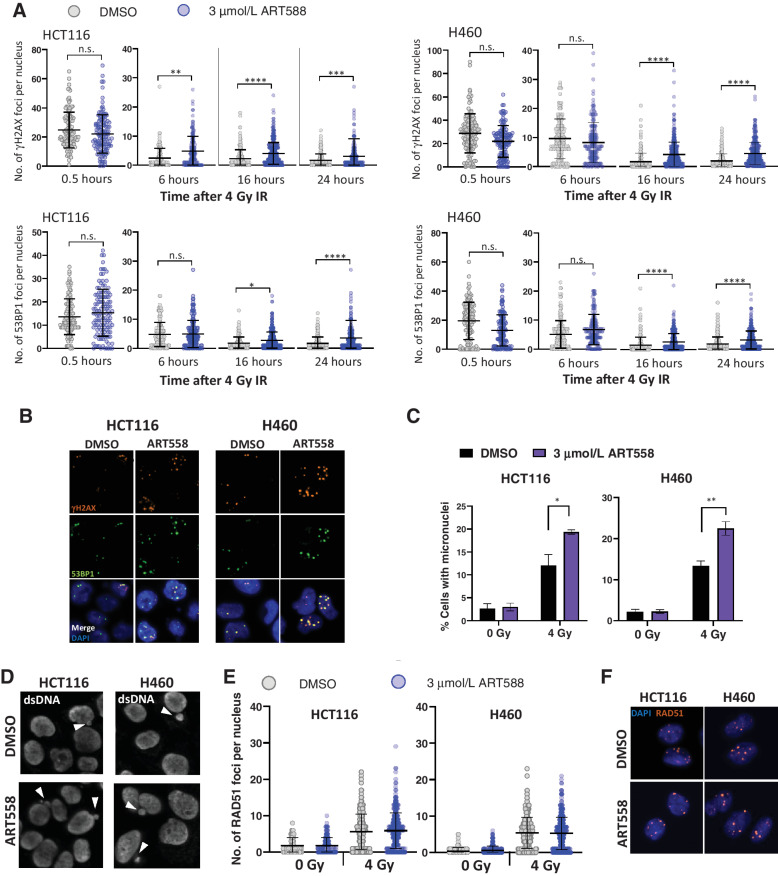
ART558 treatment leads to increased residual IR-induced DNA damage foci. **A,** γH2AX and 53BP1 foci in HCT116 and H460 cells treated with 3 μmol/L ART558 and 4 Gy IR, assessed at the indicated times points after IR. **B,** Representative images from irradiated cells from the experiment described in **A**, at 16 hours after IR. **C,** Micronuclei in HCT116 and H460 cells treated with 3 μmol/L ART558 and 4 Gy IR, assessed 48 hours after IR. Data points indicate the mean ± SD from triplicate wells and graphs are representative from three separate experiments. **D,** Representative images from irradiated cells from the experiment described in **C**. Micronuclei are indicated with arrow heads. dsDNA, double-stranded DNA. **E,** RAD51 foci in HCT116 and H460 cells treated with 3 μmol/L ART558 and 4 Gy IR, assessed 6 hours after IR. **F,** Representative images from irradiated cells from the experiment described in **E**. Lines and error bars in **A** and **E** correspond to average ± SD representative from three independent experiments, and statistical significance was calculated using nonparametric one-way ANOVA (Kruskal–Wallis) with Dunn correction for multiple comparisons (*, *P* < 0.05; **, *P* < 0.01; ***, *P* < 0.001; ****, *P* < 0.0001).

To clarify whether the radiosensitizing effect of ART558 is exerted through an impairment in DSB repair, we assessed changes in both phosphorylated H2AX (γH2AX) and the NHEJ factor 53BP1. As expected, no significant induction of γH2AX or 53BP1 foci was observed in non-irradiated cells treated with ART558 (Supplementary Fig. S3A). In contrast, we consistently observed a higher number of both γH2AX and 53BP1 foci in ART588-treated cells at 16 and 24 hours, but not at 0.5 hours after IR, as compared with DMSO-treated cells (Fig. 2A and B). These suggest that Polθ inhibition by ART558 does not result in enhanced DSB induction at the time of radiation, but in a higher number of residual DSBs due to impaired DNA damage repair. Moreover, we observed a higher proportion of cells with micronuclei in cells treated with ART558 (Fig. 2C and D), which can be attributed to a higher incidence of chromosomal aberrations caused by Polθ inhibition (32).

Polθ has been shown to directly antagonize the binding of the HR repair factor RAD51 to ssDNA (7) and, accordingly, Polθ depletion has previously been shown to increase the formation of RAD51 foci in HR-deficient cells or after treatment with DSB-inducing agents, including IR (7, 20, 33). However, we did not observe any differences in the induction of RAD51 foci at 6 hours after radiation—when RAD51 foci number are at their peak (34)—in HCT116 or H460 cells treated with ART558 (Fig. 2E and F) or upon siRNA-mediated *POLQ* depletion (Supplementary Fig. S3B).

### Effect of ART558 under hypoxic conditions

**Figure 3.**
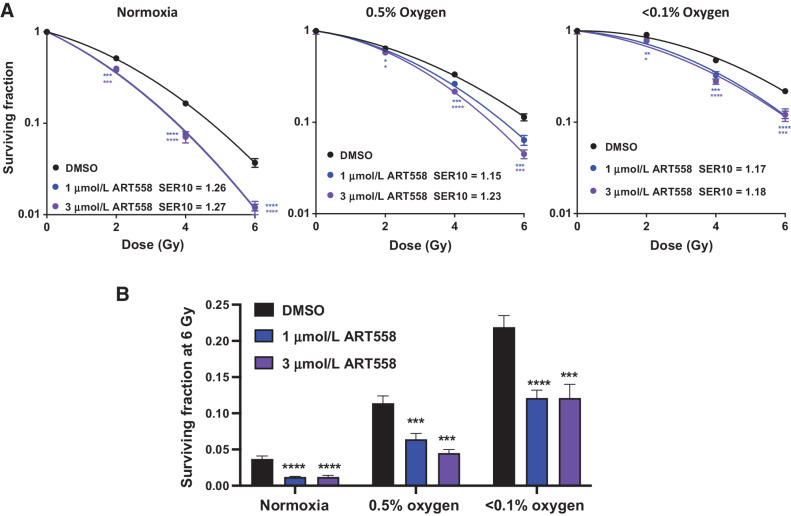
ART558-mediated radiosensitization under hypoxic conditions. **A,** Clonogenic survival of H460 cells irradiated upon hypoxia (0.5% and <0.1% oxygen). For OERs, see Supplementary Fig. S4. The 6 Gy data points are replotted in a bar graph in **B** to allow better visual comparison between the different treatment arms (*, *P* < 0.05; **, *P* < 0.01; ***, *P* < 0.001; ****, *P* < 0.0001).

Hypoxic cells can be over three times more radioresistant than normoxic cells (35, 36) and for this reason tumor hypoxia is an important mechanism of radiotherapy resistance. Therefore, we tested ART558 combined with IR at two low oxygen concentrations: 0.5% and <0.1% oxygen. The results confirmed that hypoxia induces significant resistance to IR [oxygen enhancements ratios (OER) of 1.31 and 1.54 for 0.5% and <0.1% oxygen, respectively: Supplementary Fig. S4]. Nonetheless, we found that even under severe hypoxia (<0.1% oxygen), ART558 confers significant radiosensitization, with SER_10_ values just slightly lower than those in normoxic conditions (Fig. 3A and B).

### Polθ inhibition combined with radiation is well tolerated and leads to reduced tumor growth *in vivo*

**Figure 4.**
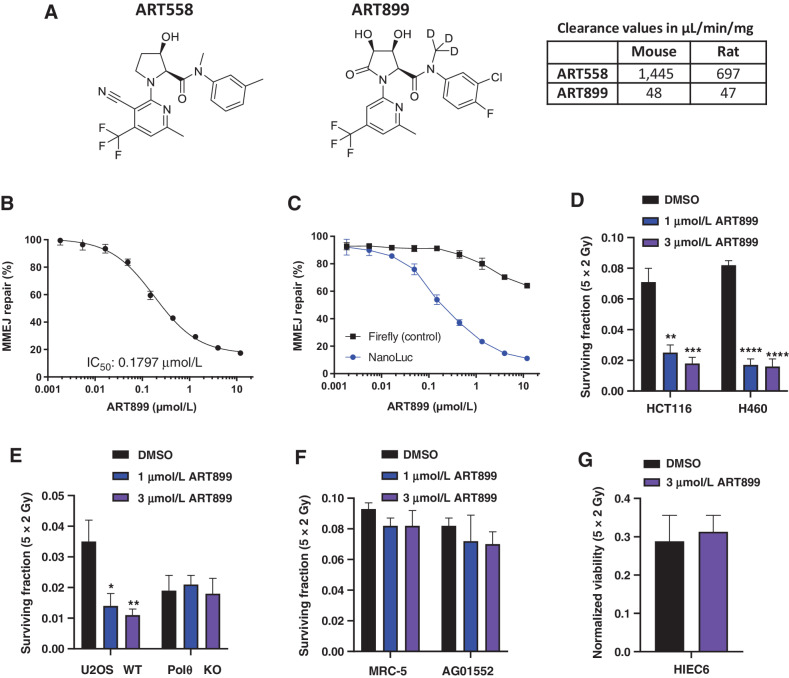
Characterization of ART899 as a specific and potent Polθ inhibitor with improved stability. **A,** Chemical structures of the Polθ inhibitors ART558 and ART899. The table shows the *in vitro* intrinsic clearance values of ART558 and ART899 after exposure to rat and mouse liver microsomes. **B,** Nano-luciferase MMEJ assay showing ART899-mediated inhibition of MMEJ activity in HEK-293 cells. The nano-luciferase readings were normalized to control luciferase (firefly) readings, and these were then normalized to DMSO. Data points show the mean ± SEM of four technical replicates; representative of two independent experiments. **C,** Confirmation of MMEJ assay specificity. Same experiment described in **B** but showing both the nanoluc and firefly readings normalized to their own DMSO reading, confirming negligible inhibition by ART899 of the control firefly luciferase signal. **D,** Clonogenic survival of HCT116 and H460 cells treated with ART899. Graphs show the surviving fraction after 5 × 2 Gy IR. **E,** Confirmation of ART899 specificity in U2OS WT and Polθ KO cells. Cells were treated as described in **D**. **F,** Effect of ART899 in noncancerous cells. MRC-5 and AG01552 fibroblasts were treated as described in **D**. The effect of ART899 in unirradiated cells from **D** to **F** is shown in Supplementary Fig. S5A. Graphs shown in **D** to **F** correspond to average ± SD from triplicate wells (representative from three separate experiments; *, *P* < 0.05; **, *P* < 0.01; ***, *P* < 0.001; ****, *P* < 0.0001). **G,** Viability of HIEC-6 cells treated with ART899 and irradiated with 5 × 2 Gy, as determined by the alamar blue assay 8 days after the first IR fraction. Graph shows the viability normalized to unirradiated controls (Supplementary Fig. S5C); representative from three independent experiments.

Next, we went on to determine the efficacy of Polθ inhibition in combination with radiation *in vivo*. As ART558 has been previously shown to be unsuitable for *in vivo* treatment due to poor metabolic stability (22), we utilized an optimized derivative, ART899 (Fig 4A). ART899 is a deuterated form of ART812, the ART558 derivate shown to have efficacy in BRCA1/SHLD2-deficient tumor xenograft studies (22). Clearance values obtained using microsome stability assays showed that ART899 has a greatly improved metabolic stability compared with ART558 (Fig 4A). To confirm that ART899 is an effective and specific Polθ inhibitor, we tested its activity in *in vitro* assays. First, we measured the inhibition of cellular MMEJ activity in a luciferase assay in HEK293 cells, a system previously used to demonstrate ART558 activity (22). This showed that ART899 has a cellular IC_50_ of approximately 180 nmol/L (Fig. 4B and C), which is comparable to the value previously reported for ART558 (IC_50_ = 150 nmol/L; ref. 22). We then deployed the assay in a panel of relevant cell lines and further confirmed that ART899 IC_50_s are similar to those of ART558 (Supplementary Table S1).

Because our earlier results have shown that ART558 is more effective when combined with multiple IR doses rather than a single IR dose (Fig. 1), we investigated the radiosensitizing efficacy of ART899 in combination with fractionated IR (5 × 2 Gy). This showed that ART899 effectively radiosensitizes HCT116 and H460 cells at both 1 and 3 μmol/L (Fig. 4D). At the highest ART899 concentration (3 μmol/L), the decrease in survival in H460 cells was 5-fold compared with IR alone; for HCT116 this factor was 4-fold. Similar to ART558, we did not observe any effect of ART899 in the absence of irradiation (Supplementary Fig. S5A).

Specificity for Polθ was further confirmed by showing a lack of radiosensitization in U2OS Polθ KO cells (Fig. 4E) and si*POLQ*-treated HCT116 and H460 cells (Supplementary Fig. S5B). Prior to starting *in vivo* studies, it was important to confirm that ART899 does not radiosensitize noncancerous cells. As shown in Fig. 4F, the clonogenic capacity of the human fibroblast lines MRC5 and AG01552 did not further decrease with ART899 in combination with 5×2 Gy IR. We also tested ART899 in combination with fractionated IR in the noncancerous epithelial cell line HIEC-6. As HIEC-6 cells are unable to form colonies, we assessed their proliferative capacity using the alamar blue assay, which showed that HIEC-6 cells were not radiosensitized by ART899 (Fig. 4G), further demonstrating the tumor specificity of this Polθ inhibitor.

**Figure 5.**
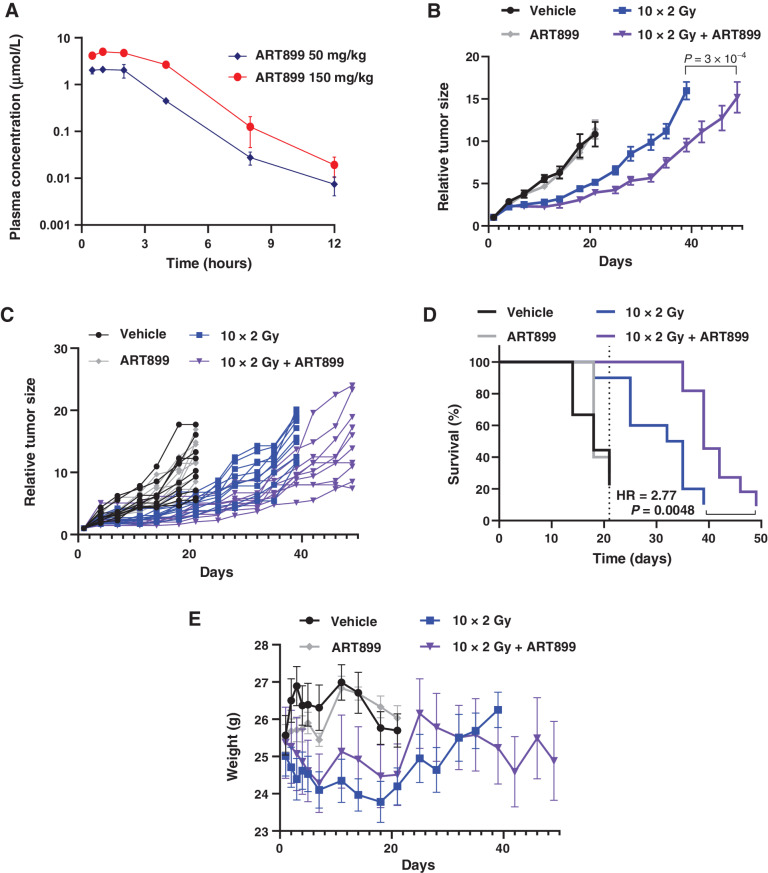
Polθ inhibitor ART899 combined with radiation causes significant tumor growth delay *in vivo* and is well tolerated. **A,** ART899 plasma concentration following oral dosage of ART899 at 50 or 150 mg/kg. Mouse plasma samples (*n* = 3 per treatment group) were collected at 30 minutes, 1, 2, 4, 8, and 12 hours after last dose. **B–E,** HCT116 tumor-bearing mice treated with 150 mg/kg Polθ inhibitor ART899 twice daily for 12 days and/or 10 × 2 Gy (days 1–5 and 8–12). Vehicle (*n* = 9); ART899 (*n* = 10); 10 × 2 Gy + vehicle (*n* = 10); 10 × 2 Gy + ART899 (*n* = 10). **B,** Mean ± SEM relative tumor size. *P* value from mixed effect model and Dunnett post-test. Comparison of tumor size at the latest common timepoint for 10 × 2 Gy versus 10 × 2 Gy + ART899 are shown in Supplementary Fig. S6A. **C,** Individual mouse graphs. **D,** Kaplan–Meier plot for a tumor size threshold of 1,000 mm^3^. HR: Hazard ratio (hazard rate of IR arm / hazard rate of IR + ART899 arm); *P* value from the log-rank (Mantel-Cox) test comparing IR alone and IR + ART558. The median time to a tumor size of 1,000 mm^3^ for the IR + ART899 arm versus the IR arm and the corresponding ratio are shown in Supplementary Fig. S6B. **E,** Average mouse weight ± SD from all treatment groups over time. Individual mouse weights are shown in Supplementary Fig. S6C.

We then confirmed that ART899 possesses a good *in vivo* pharmacokinetic profile, with a plasma concentration ≥1 μmol/L for several hours after oral administration in mice (Fig. 5A). Finally, we tested ART899 in mice bearing HCT116 subcutaneous xenografts. The combination of ART899 treatment with fractionated irradiation (10 × 2 Gy) significantly improved tumor growth delay compared with radiation alone (Fig. 5B–D; Supplementary Figs. S6A and S6B). Importantly, the combination treatment was well tolerated with mice displaying no weight loss, or other signs of distress or treatment-related adversity, compared with IR alone, during the course of the experiment and as per necropsy at the experiment end (Fig. 5E; Supplementary Figs. S6C and Materials and Methods). Together, these data show that these novel Polθ inhibitors are effective tumor-selective radiosensitizers in both *in vitro* and *in vivo* settings.

## Discussion

Here, we demonstrate the radiosensitizing effect of novel Polθ inhibitors ART558 and ART899. ART558 was previously shown to have a potent stand-alone antitumor effect in cancer cells with defects in HR and the Shieldin complex, and in combination treatment with PARP inhibitors (22). We now show that ART558 is also able to significantly radiosensitize HR-proficient cells in a Polθ-specific fashion. In addition, we report for the first time ART899, a novel derivative of ART558 with increased stability and efficacy against Polθ *in vivo*. We confirmed that ART899 specifically inhibits Polθ MMEJ activity and that specifically radiosensitizes tumor cells with no effect on some noncancerous cells, consistent with the low or absent expression of Polθ in normal tissues and overexpression in many cancer cells (4, 5, 21).

We also report here a higher proportion of residual IR-induced γH2AX and 53BP1 foci upon pharmacologic inhibition of Polθ linked to increased formation of IR-induced micronuclei, which strongly suggests that the mechanism whereby our Polθ inhibitors radiosensitize cancer cells is by an impairment in DSB repair, which leads to lethal chromosomal rearrangements (32). Our finding that neither Polθ inhibition nor siRNA-mediated Polθ depletion lead to a higher number of RAD51 foci in HCT116 and H460 cells following irradiation is in contrast with earlier findings in other HR-proficient cell lines (7, 20), suggesting that the impact of Polθ impairment on the recruitment of RAD51 to DSBs might be cell context dependent.

Importantly, we show that Polθ inhibition potently radiosensitizes cancer cells exposed to multiple fractions of radiation, both *in vitro* and *in vivo*. The translational relevance of this is that, in a clinical setting, radiotherapy is commonly administered in multiple doses—that is, fractionated radiotherapy—which minimizes toxicity to normal tissues. In line with the known cell-cycle selectivity of the MMEJ repair pathway (31), we confirmed that cells traversing S phase are more sensitive to Polθ inhibition than cells in G_1_ phase. Because cancer cells are known to transit through the cell cycle during the time between IR fractions—which increases the likelihood of cancer cells being irradiated at more radiosensitive cell-cycle phases (30)—this finding provides mechanistic evidence supporting the advantage of delivering a fractionated radiotherapy regime when using Polθ inhibitors. In addition, we show for the first time that Polθ inhibition can radiosensitize tumor cells under hypoxia, a common feature of solid tumors that confers radioresistance (36). Our results therefore position Polθ inhibition as a promising strategy to improve the efficacy of radiotherapy regardless of the presence of tumor hypoxia. Further investigation is needed to establish whether this family of Polθ inhibitors can achieve high enough concentrations in tumor hypoxic regions—as these regions display limited perfusion—as to induce effective radiosensitization.

Finally, we show here that combining ART899 with fractionated IR in xenograft models results in significantly improved tumor growth delay and is well tolerated. Polθ inhibition is being currently tested in a first-in-human clinical trial (NCT04991480), which is evaluating the safety and activity of Polθ inhibition in patients with solid tumors. This clinical trial could open new clinical avenues to explore the therapeutic modality we have discovered in this work. Our study broadens the potential therapeutic application of Polθ inhibition beyond its use as a therapy for tumors with defects in HR repair, with the promise to improve the efficacy of radiotherapy.

## Supplementary Material

Supplementary Figure S1 Distribution of POLQ mRNA levels across cell lines from CCLE

Supplementary Table S1 Luciferase-based MMEJ assay in a panel of cancer cell lines.

Supplementary Figure S2 Cell cycle-dependent effects of ART558 in combination with IR

Supplementary Figure S3 Accompanies Figure 2 (ART558 treatment leads to increased residual IR-induced DNA damage foci)

Supplementary Figure S4 Accompanies Figure 3 (Effect of ART558 under hypoxic conditions)

Supplementary Figure S5 Accompanies Figure 4 (Characterization of ART899 as a specific and potent Polθ inhibitor with improved stability)

Supplementary Figure S6 Accompanies Figure 5 (ART899 combined with radiation causes significant tumor growth delay in vivo and is well tolerated)
