# Supplementary material for: Small-Molecule Polθ Inhibitors Provide Safe and Effective Tumor Radiosensitization in Preclinical Models
Source: Clin Cancer Res. 2023 Jan 23;29(8):1631–42. doi: 10.1158/1078-0432.CCR-22-2977 (PMC10102842; doi:10.1158/1078-0432.CCR-22-2977)
Supplement: Supplementary Figure S1 — Distribution of POLQ mRNA levels across cell lines from CCLE [file ccr-22-2977_supplementary_figure_s1_suppfs1.pdf]

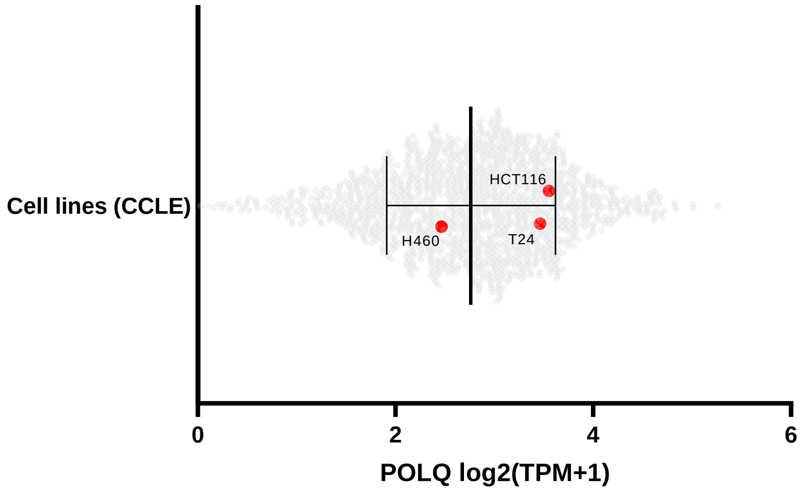

Supplementary Figure S1. Distribution of POLQ mRNA levels, expressed as log2 of the Transcript Count Per Million (log2(TPM + 1), across the 1406 cell lines from the *Cancer Cell Line Encyclopaedia* (CCLE) (22Q2 dataset; mean +/- standard deviation) . The distribution of the cancer cell lines used in the CFAs shown in Figure 1B is highlighted in red.
