# Supplementary material for: Small-Molecule Polθ Inhibitors Provide Safe and Effective Tumor Radiosensitization in Preclinical Models
Source: Clin Cancer Res. 2023 Jan 23;29(8):1631–42. doi: 10.1158/1078-0432.CCR-22-2977 (PMC10102842; doi:10.1158/1078-0432.CCR-22-2977)
Supplement: Supplementary Figure S2 — Cell cycle-dependent effects of ART558 in combination with IR [file ccr-22-2977_supplementary_figure_s2_suppfs2.pdf]

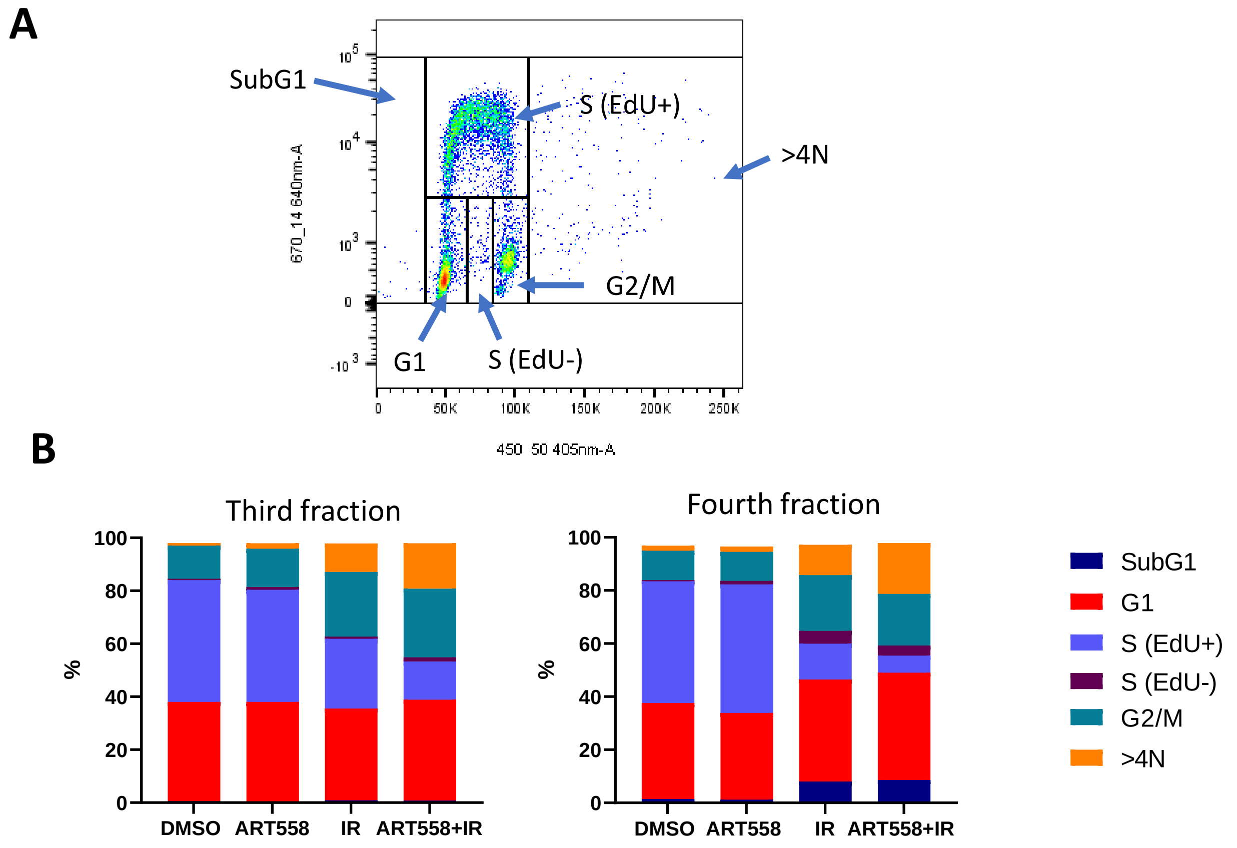


**Supplementary Figure S2.** Cell cycle-dependent effects of ART558 in combination with IR. HCT116 cells were treated with either DMSO or 10 µM ART558 and exposed to fractionated IR (2 Gy every 24 hours). DMSO and ART558 were added 1 h before the first radiation fraction and kept throughout the duration of the experiment. **A)** Representation of the FACS gating strategy to measure the different cell cycle phases. The plot represents a vehicle treated control at 0 h timepoint. S (EdU+) subpopulation corresponds to actively replicating S phase cells. >4N subpopulation corresponds to polyploid cells. **B**) Cell cycle distribution of cells collected 24 hours after the second and the third IR fractions (i.e., at the time of the third and fourth fractions in the clonogenic survival experiments, respectively). Results are representative of two independent experiments.
