# Supplementary material for: Small-Molecule Polθ Inhibitors Provide Safe and Effective Tumor Radiosensitization in Preclinical Models
Source: Clin Cancer Res. 2023 Jan 23;29(8):1631–42. doi: 10.1158/1078-0432.CCR-22-2977 (PMC10102842; doi:10.1158/1078-0432.CCR-22-2977)
Supplement: Supplementary Figure S3 — Accompanies Figure 2 (ART558 treatment leads to increased residual IR-induced DNA damage foci) [file ccr-22-2977_supplementary_figure_s3_suppfs3.pdf]

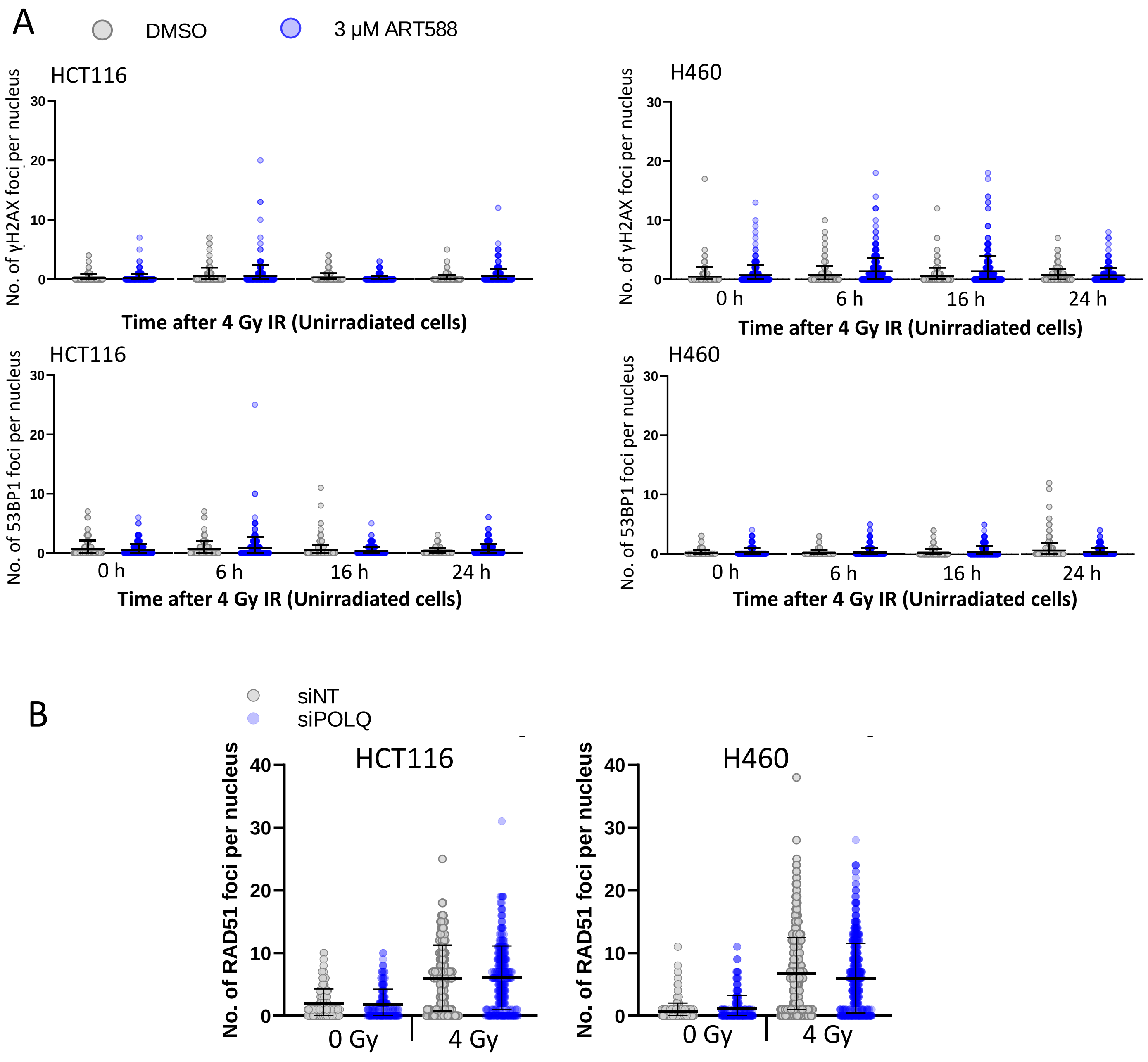

Supplementary Figure S3. Accompanies Figure 2 (ART558 treatment leads to increased residual IR-induced DNA damage foci). (**A**) Number of γH2AX and 53BP1 foci (mean +/- standard deviation) in unirradiated HCT116 and H460 cells treated with DMSO or 3µM ART558 (from experiment shown in Figure 2A. (**B**) Number of RAD51 foci (mean +/- standard deviation) in HCT116 and H460 cells treated with either non-targeting siRNA (siNT) or an siRNA targeting POLQ (siPOLQ) 6 h after 4 Gy IR. (from experiment shown in Figure 2E)
