# Supplementary material for: Small-Molecule Polθ Inhibitors Provide Safe and Effective Tumor Radiosensitization in Preclinical Models
Source: Clin Cancer Res. 2023 Jan 23;29(8):1631–42. doi: 10.1158/1078-0432.CCR-22-2977 (PMC10102842; doi:10.1158/1078-0432.CCR-22-2977)
Supplement: Supplementary Figure S4 — Accompanies Figure 3 (Effect of ART558 under hypoxic conditions) [file ccr-22-2977_supplementary_figure_s4_suppfs4.pdf]

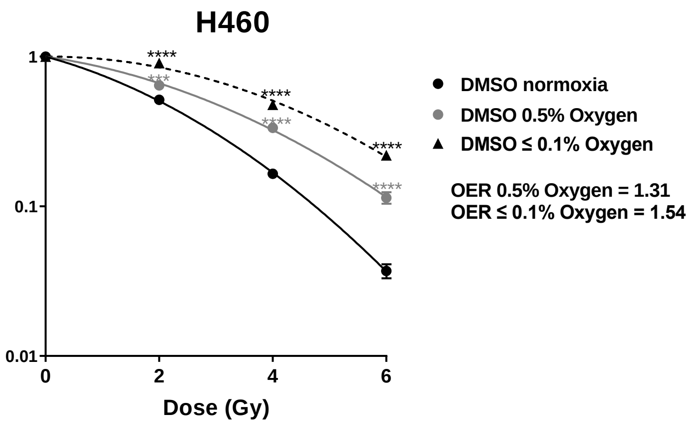

Supplementary Figure S4. Accompanies Figure 3 (Effect of ART558 under hypoxic conditions). Clonogenic survival graph comparing control (DMSO) curves from Figure 3A, confirming resistance to IR induced by hypoxia. OER: oxygen enhancement ratios.*** $p<0.001$; **** $p<0.0001$.
