# Supplementary material for: Small-Molecule Polθ Inhibitors Provide Safe and Effective Tumor Radiosensitization in Preclinical Models
Source: Clin Cancer Res. 2023 Jan 23;29(8):1631–42. doi: 10.1158/1078-0432.CCR-22-2977 (PMC10102842; doi:10.1158/1078-0432.CCR-22-2977)
Supplement: Supplementary Figure S5 — Accompanies Figure 4 (Characterization of ART899 as a specific and potent Polθ inhibitor with improved stability) [file ccr-22-2977_supplementary_figure_s5_suppfs5.pdf]

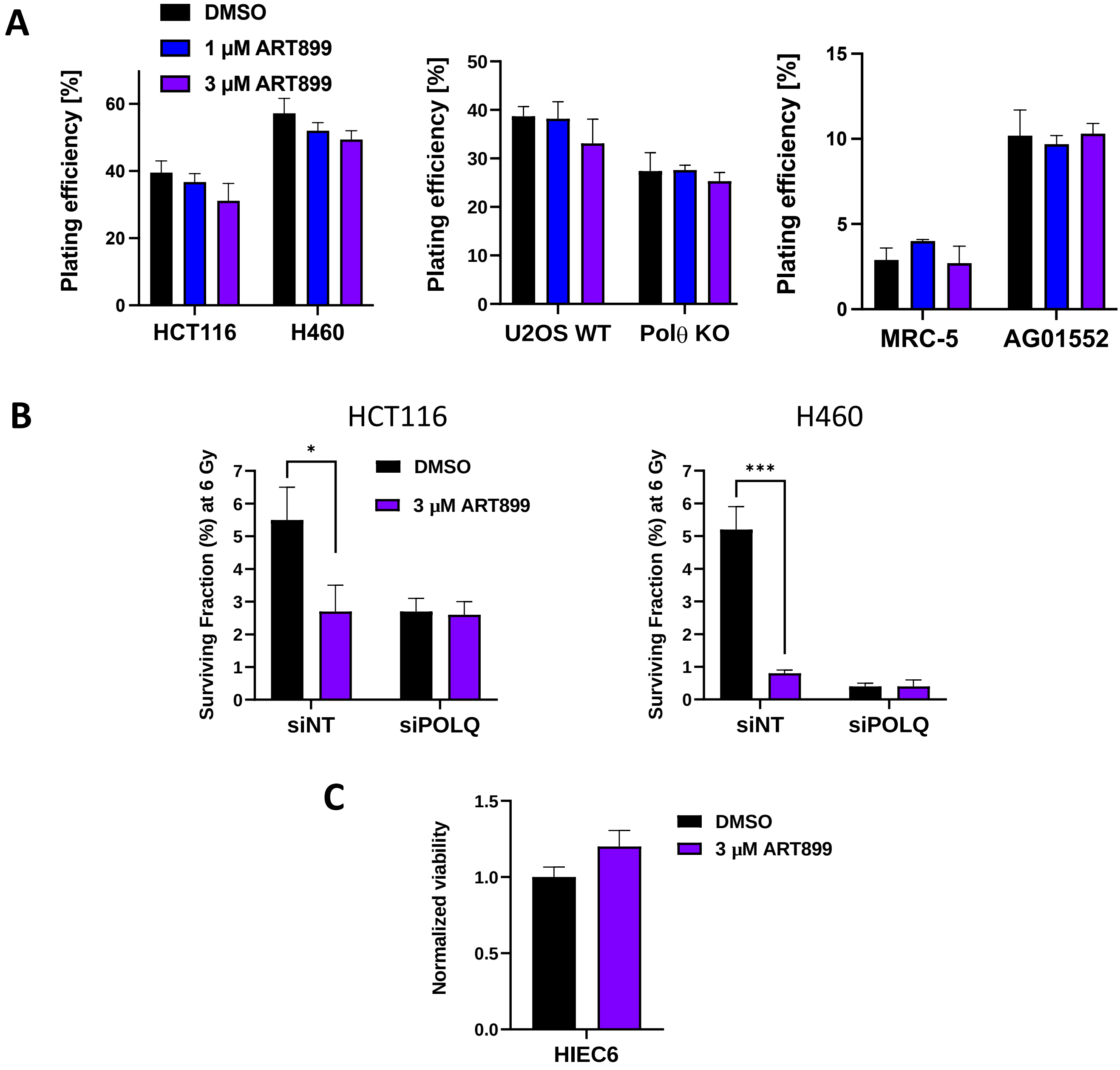

Supplementary Figure S5. Accompanies Figure 4 (Characterization of ART899 as a specific and potent Polθ inhibitor with improved stability). (**A**) Clonogenic survival of HCT116 and H460 cells transfected with either a non-targeting siRNA (siNT) or an siRNA targeted against POLQ (siPOLQ) and treated with 3 μM ART899 and 6 Gy IR. Bar graphs show the surviving fraction at 6 Gy. (**B**) Effect of ART899 in unirradiated cells from colony formation assays described in Figure 4D-F. (**C**) Effect of ART899 in unirradiated cells from Alamar blue assay described in Figure 4G. Graph show the viability normalized to DMSO-treated cells).
