# Supplementary material for: Small-Molecule Polθ Inhibitors Provide Safe and Effective Tumor Radiosensitization in Preclinical Models
Source: Clin Cancer Res. 2023 Jan 23;29(8):1631–42. doi: 10.1158/1078-0432.CCR-22-2977 (PMC10102842; doi:10.1158/1078-0432.CCR-22-2977)
Supplement: Supplementary Figure S6 — Accompanies Figure 5 (ART899 combined with radiation causes significant tumor growth delay in vivo and is well tolerated) [file ccr-22-2977_supplementary_figure_s6_suppfs6.pdf]

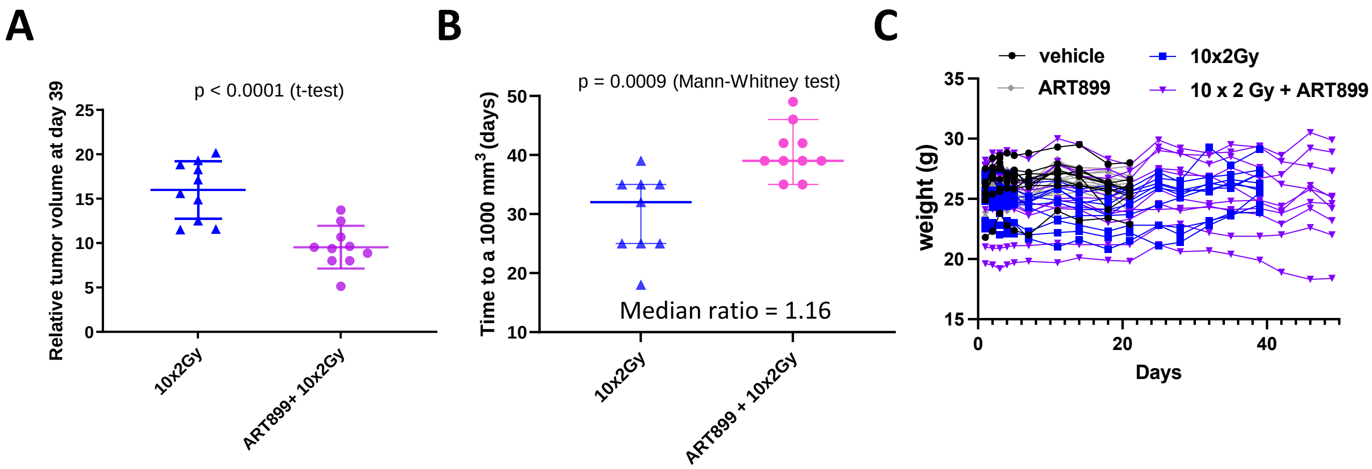

Supplementary Figure S6. Accompanies Figure 5 (ART899 combined with radiation causes significant tumor growth delay *in vivo* and is well tolerated). (**A**) Comparison of tumor size at day 39, from Figure 5B. (**B**) Median time to a tumor size of 1000 mm^3 (error bars correspond to 95% confidence interval). The ratio of the median time for the ART899 + 10 x 2 Gy arm to the median time of the 10 x 2 Gy arm is shown. From data shown in Figure 5D. (**C**) Individual mouse graphs from Figure 5E.
